# A General Biosensing Strategy Based on Cascade Amplification for Enhanced HIV Detection Sensitivity

**DOI:** 10.34133/bmef.0139

**Published:** 2025-07-02

**Authors:** Yirui Zhang, Jieyu Yan, Wangheng Hou, Qian Gao, Tao Zhang, Yan Gao, Jingwen Li, Kun Han

**Affiliations:** ^1^ School of Biomedical Engineering (Suzhou), Division of Life Sciences and Medicine, University of Science and Technology of China, Hefei 230026, China.; ^2^ CAS Key Lab of Bio-Medical Diagnostics, Suzhou Institute of Biomedical Engineering and Technology, Chinese Academy of Science, Suzhou 215163, China.; ^3^ NMPA Key Laboratory for Research and Evaluation of Infectious Disease Diagnostic Technology, School of Public Health, Xiamen University, Xiamen 361102, China.; ^4^ Suzhou Center for Disease Control and Prevention, Suzhou 215004, China.

## Abstract

**Objective:** Accurate and rapid detection of disease biomarkers is critical for early diagnosis, timely intervention, and effective disease management. **Impact Statement:** This strategy is exemplified through the development of 2 biosensors for detecting HIV-1 DNA and HIV-1 p24, biomarkers associated with acquired immunodeficiency syndrome (AIDS). **Introduction:** We propose a general biosensing strategy that leverages a treble signal amplification cascade, demonstrating its versatility and applicability across diverse biomolecular targets. **Methods:** The method integrates multiple amplification mechanisms to achieve unparalleled sensitivity. Initially, the hybridization of 2 aided probes with the target triggers isothermal amplification facilitated by polymerase and nicking enzyme, providing a robust preliminary signal enhancement. Repeated cycles of primer extension, nicking, and signal primer dissociation then generate multiple signal primers. These primers are further amplified via rolling circle amplification (RCA), resulting in an important secondary signal boost. Finally, the amplified products activate CRISPR-Cas12a-mediated trans-cleavage, achieving a tertiary level of signal enhancement. **Results:** This cascade amplification approach achieves remarkable sensitivity, with detection limits of 62 aM for nucleic acids and 8.48 pg/ml for proteins, positioning it as a broadly applicable framework. Clinical samples were assayed, which indicates its capability in clinical diagnosis. **Conclusion:** Beyond HIV detection, the modular design of this strategy allows adaptation for various biomarkers, showcasing its potential as a universal platform for molecular diagnostics in healthcare and research.

## Introduction

Human immunodeficiency virus (HIV), the causative agent of acquired immunodeficiency syndrome (AIDS), continues to represent one of the most pressing global health crises. HIV infection compromises the immune system by targeting CD4 helper T lymphocytes, resulting in progressive immune dysfunction and heightened susceptibility to severe opportunistic infections and malignancies [1]. Since the first cases of HIV/AIDS were reported, the disease has caused immense societal and economic burdens, with devastating impacts on healthcare systems worldwide. According to recent data from the World Health Organization (WHO), over 84.2 million individuals have been infected with HIV globally, and nearly half (48%) of these individuals have succumbed to AIDS-related illnesses [2–4]. These alarming statistics underscore the urgent need for effective HIV detection strategies that can facilitate timely diagnosis, initiate prompt therapeutic interventions, and ultimately reduce morbidity and mortality [5–10].

The accurate and early detection of HIV not only is critical for individual patient outcomes but also plays a pivotal role in controlling the spread of the virus within populations. HIV detection methods vary based on the stage of infection and include approaches targeting antigens [11,12], antibodies [13–15], CD4 cell counts [16], and viral loads [17,18]. Traditional techniques, such as polymerase chain reaction (PCR) [19,20], enzyme-linked immunosorbent assay (ELISA) [21], and Western blotting [22], have been widely adopted but face significant challenges, including labor-intensive protocols, high costs, and limited sensitivity for detecting low-level infections. These limitations hinder their broader application in resource-limited settings, where the HIV epidemic is often most severe [23].

To address these challenges, innovative signal amplification methods have emerged as promising alternatives, offering enhanced sensitivity and specificity. Enzyme-assisted amplification techniques, characterized by their rapid reaction kinetics and robust signal enhancement, have enabled the development of advanced diagnostic platforms, including fluorescence-based assays [24], colorimetric assays [25], and electrochemical tests [26]. Among these, nucleic acid amplification strategies such as catalytic hairpin assembly (CHA) [27], hybridization chain reaction (HCR) [28], loop-mediated isothermal amplification (LAMP) [29], strand displacement amplification, and rolling circle amplification (RCA) [30] have demonstrated significant promise. RCA, in particular, has gained widespread attention due to its exceptional accuracy, scalability, and ease of operation [31].

In recent years, breakthroughs in clustered regularly interspaced short palindromic repeats and CRISPR-associated proteins (CRISPR-Cas) have revolutionized the field of molecular diagnostics [32–34]. Within this framework, CRISPR-Cas12a has emerged as a powerful tool for nucleic acid detection, owing to its robust trans-cleavage activity and programmability [35–37]. When activated by the binding of CRISPR RNA (crRNA) to a double-stranded target, Cas12a exhibits indiscriminate single-stranded DNA (ssDNA) cleavage, a unique feature that has been harnessed in the design of biosensors employing fluorescence, electrochemistry, and colorimetric readouts [38–41]. These advancements have not only enhanced the sensitivity and versatility of HIV detection but also paved the way for broader applications in diagnosing other infectious diseases and genetic disorders.

As the global community continues its efforts to eradicate HIV/AIDS, the development and deployment of sensitive, cost-effective, and accessible diagnostic tools remain paramount. By addressing the limitations of conventional methods, advanced detection strategies integrating RCA and CRISPR/Cas systems offer a transformative approach to combating HIV, reducing transmission, and improving patient outcomes worldwide.

Building on recent advancements in molecular diagnostics, we propose a versatile and generalizable biosensing framework exemplified by 2 bioassays for HIV-1 detection: one targeting the conserved region of the HIV-1 gag gene and another for the HIV-1 capsid protein p24. This approach integrates RCA and CRISPR-Cas12a-assisted cascade amplification, achieving highly sensitive and specific detection. The underlying mechanism and testing procedures are designed to be modular and adaptable, highlighting the strategy’s potential for broad applicability across various targets.

In the presence of a target, 2 carefully engineered probes are brought into spatial proximity through simultaneous interactions with the target, initiating isothermal amplification. This initial step produces a substantial quantity of ssDNA, which subsequently triggers RCA amplification via hybridization with a padlock probe. RCA products then act as activators for the CRISPR/Cas12a system, whose robust trans-cleavage activity generates amplified fluorescence signals. Importantly, each step in this cascade is optimized for flexibility, enabling straightforward adaptation to detect a wide range of nucleic acid and protein biomarkers by simply reconfiguring the probe design. By leveraging the unparalleled signal amplification capabilities of RCA and the programmable, sequence-specific cleavage activity of CRISPR-Cas12a, this method provides a powerful, sensitive, and efficient platform for molecular detection. Beyond its application in HIV-1 diagnostics, the cascade amplification strategy is inherently versatile, offering a unified framework for the detection of diverse biomolecules. This generalizable design represents a significant advancement not only in AIDS detection but also in the broader field of molecular diagnostics, where rapid, accurate, and accessible detection of disease biomarkers is critical. The integration of RCA and CRISPR-Cas12a into a single workflow underscores the modularity and scalability of this approach. Whether applied to viral, bacterial, or even cancer biomarkers, this biosensing platform has the potential to revolutionize diagnostic methodologies by providing a universally adaptable and highly sensitive solution for detecting critical molecular targets. By addressing current limitations in sensitivity, specificity, and operational complexity, this strategy holds immense promise for advancing diagnostic technologies across healthcare and research domains.

## Results

### Design principles of HIV-1 DNA detection

**Fig. 1.**
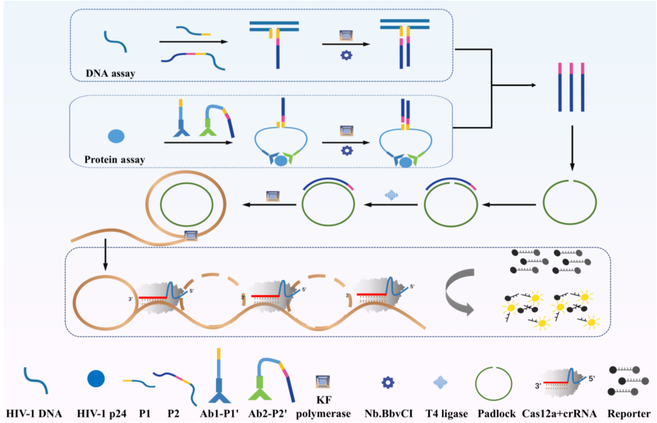
Schematic Illustration of HIV-1 DNA and HIV-1 p24 detection.

The design of our proposed biosensor, as illustrated in Fig. 1, exemplifies a generalizable approach to nucleic acid detection, utilizing a cascade amplification strategy that can be readily adapted for diverse biomarkers. In this work, we selected the highly conserved region of the HIV-1 gag gene as the target analyte to demonstrate the effectiveness of this strategy. The sensing platform integrates 3 key processes: target-induced binding and isothermal amplification, RCA, and fluorescence signal detection mediated by CRISPR/Cas12a. Two hybridization probes, P1 and P2, were specifically designed with a short 5-nucleotide complementary region to prevent nonspecific binding in the absence of the target, ensuring high specificity and minimal background noise. Upon the presence of the target, the probes hybridize to form a 3-way junction, triggering a primer extension reaction catalyzed by a polymerase to produce ssDNA (primer). This primer then binds to a pre-designed padlock probe, initiating the RCA process in the presence of KF (Klenow Fragment, Exo-) polymerase and deoxynucleotide triphosphates (dNTPs). RCA generates long repeating DNA sequences, which serve as potent molecular activators. These activators are subsequently recognized by CRISPR/Cas12a via complementary pairing with crRNA, activating the enzyme’s robust trans-cleavage activity and leading to the cleavage of ssDNA reporters, thereby generating measurable fluorescent signals.

### Characterization and optimization of HIV-1 DNA detection

The versatility and adaptability of this design are underscored by its modular nature, allowing for straightforward reconfiguration of the probes to target different nucleic acid sequences. This adaptability ensures that the system is not limited to HIV-1 detection but can be expanded to a broad range of applications in molecular diagnostics, including the detection of viral, bacterial, or cancer-related biomarkers. Validation experiments using polyacrylamide gel electrophoresis (PAGE) confirmed the feasibility and integrity of each amplification step. For instance, successful primer extension and isothermal amplification were observed only when all necessary components, including the target and Nb.BbvCI, were present, highlighting the system’s specificity and robustness (Fig. S2A). This biosensor design, which integrates the precision of CRISPR/Cas12a and the amplification power of RCA, demonstrates a significant advancement in biomolecular detection technology. Its simplicity, sensitivity, and adaptability make it a promising universal platform for diagnostics, particularly in resource-limited settings where efficient and reliable detection methods are critically needed.

**Fig. 2.**
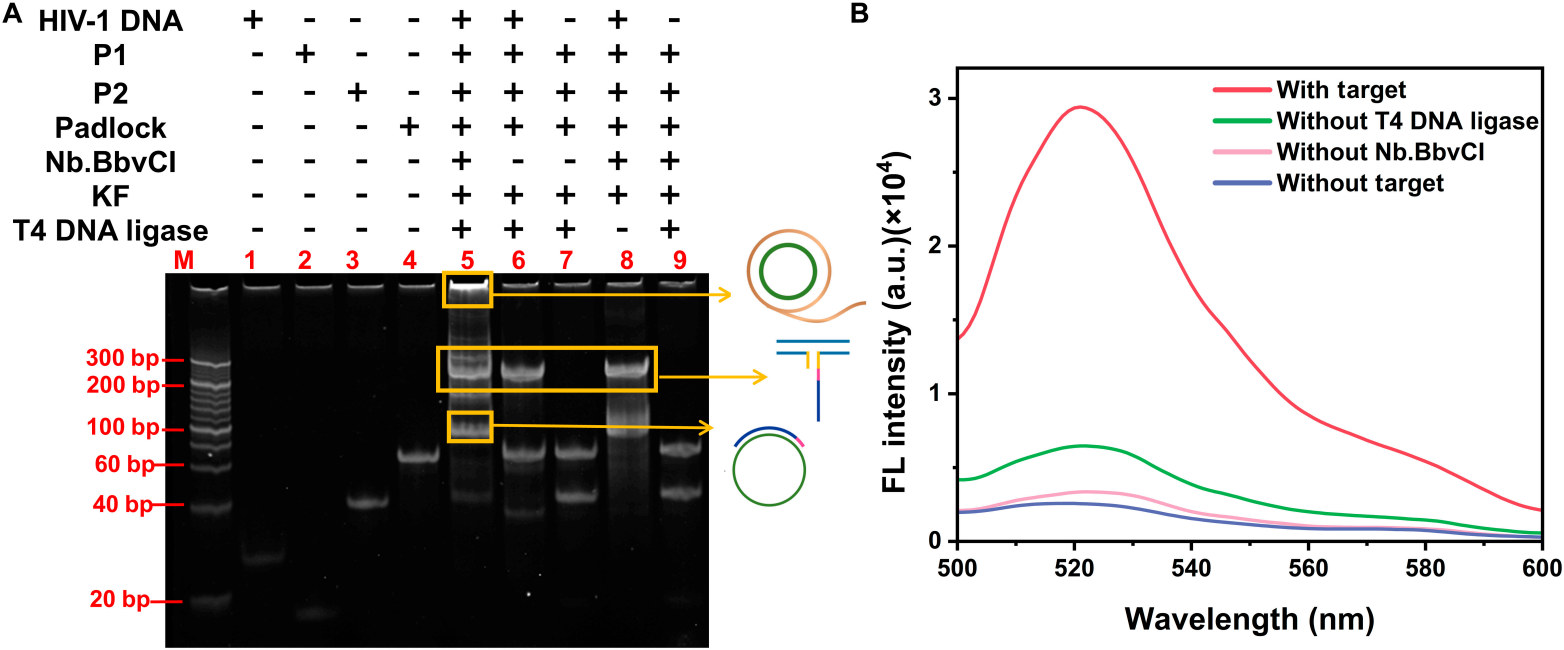
The feasibility was characterized by 15% of PAGE and fluorescence signals. (A) From left to right: lane 1, HIV-1 DNA; lane 2, P1; lane 3, P3; lane 4, padlock; lane 5, complete sample; lane 6, sample without Nb.BbvCI; lane 7, sample without HIV-1 DNA and Nb.BbvCI; lane 8, sample without T4 DNA ligase; lane 9, sample without target. (B) From top to bottom: sample with target, sample with target and Nb.BbvCI but without T4 DNA ligase, sample with target and T4 DNA ligase but without Nb.BbvCI, and sample without target.

The successful initiation of RCA amplification was confirmed by the appearance of elongated ssDNA products in lane 5 of Fig. S2B, demonstrating the primer and padlock’s ability to drive the RCA process. In contrast, controls lacking either KF polymerase or T4 DNA ligase (lanes 4 and 5) failed to produce RCA products, validating the necessity of these components for amplification. Further confirmation of HIV-1 DNA detection feasibility was obtained using 15% PAGE (Fig. 2A). Lane 5 of the completed reaction system displayed RCA amplification products, characterized by the formation of a 3-way junction and circular structure bands, confirming successful cyclic enzymatic cleavage and RCA amplification. In lane 9, the absence of the target prevented the formation of the triphasic junction and primer generation, resulting in no circular structures or amplification products. Similarly, lane 6, which lacked endonuclease despite the presence of the target, failed to produce amplification products, indicating that cyclic enzymatic cleavage and primer generation are critical for padlock probe activation. In lane 7, the absence of both the target and KF polymerase resulted in no 3-way junction or amplification. Lane 8, while containing the target and triphasic junction, failed to form closed-loop structures and amplification products due to the absence of T4 DNA ligase. These findings conclusively demonstrate that the observed amplification is specific to the RCA process rather than simple extension amplification. Supporting fluorescence spectra (Fig. 2B) further validate the system’s capability for sensitive HIV-1 DNA detection.

To optimize the system for maximum performance, we first examined the number of complementary bases between P1 and P2. Excessive base pairing could lead to background signals due to nonspecific binding, while insufficient pairing could hinder effective hybridization. Variants with 4, 5, and 6 complementary bases were tested (Fig. S3). Without the target, 6P1/6P2 triggered a primer extension reaction (lane 3), whereas no extension was observed for 4P1/4P2 in the presence of the target (lane 4). Isothermal amplification was successful with both 5P1/5P2 and 6P1/6P2 (lanes 5 and 6), with 5P1/5P2 selected for further experiments due to its balance between specificity and efficiency.

The reaction times and concentrations of critical components, including Nb.BbvCI, T4 DNA ligase, and the Cas12a system, were systematically optimized to enhance amplification efficiency. Optimal reaction times were identified as 45 min for Nb.BbvCI, 40 min for T4 DNA ligase, and 40 min for the Cas12a system (Fig. S4A to C). Optimal concentrations were determined to be 1 U/μl for Nb.BbvCI and 2 U/μl for KF polymerase (Fig. S4D and E).

### Analytical performance of HIV-1 DNA detection

**Fig. 3.**
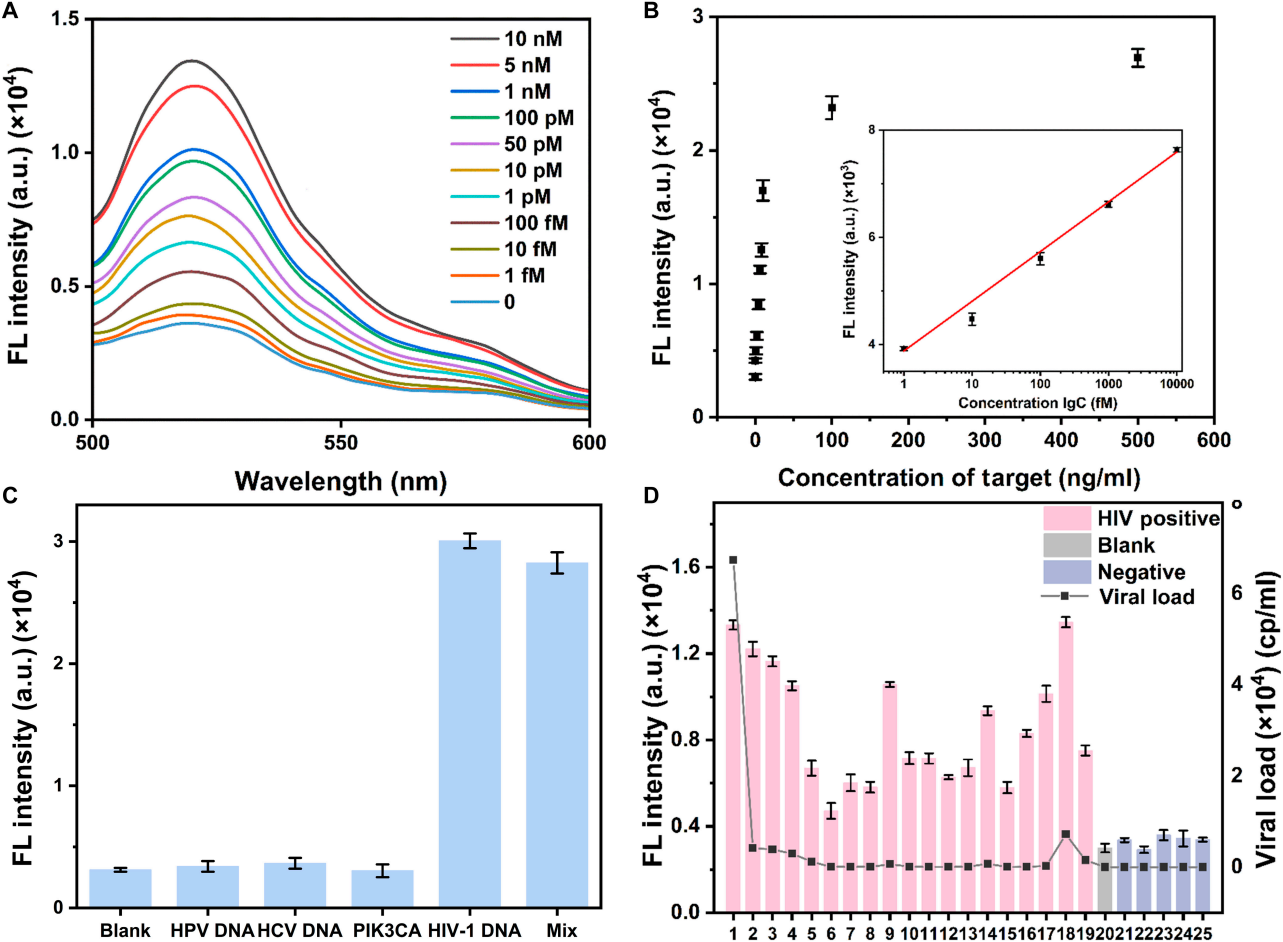
(A) Spectra of fluorescence for various concentrations of the target. (B) Correlation between efficiency of energy transfer in fluorescence and varying concentrations of HIV-1 DNA. The inner illustration shows linear relationship between the fluorescence signal and the logarithm of HIV-1 DNA concentration from 0 to 10 pM. (C) Specificities for different DNA fragment [human papillomavirus DNA (HPV DNA), hepatitis C virus DNA (HCV DNA), a mutated gene in breast cancer (PIK3CA), HIV-1 DNA, and a mixture (HIV-1 DNA + per interfering substance)]. (D) Detection of clinical samples. Error bars mean SD, and every experiment was adopted 3 times.

Under these optimized conditions, the system’s sensitivity was evaluated by detecting HIV-1 DNA across various concentrations. As shown in Fig. 3A, fluorescence intensity increased with rising HIV-1 DNA concentrations from 0 to 10 nM. A strong linear relationship was observed between fluorescence intensity and the logarithm of HIV-1 DNA concentration over a range from 1 fM to 10 pM (Fig. 3B), with a regression equation of *y* = 927.680 lgC + 6,663.741 and a correlation coefficient of 0.997. The limit of detection (LOD) was calculated to be 62 aM using the 3σ rule, underscoring the system’s remarkable sensitivity. Comparisons with other biosensors for HIV-1 DNA detection (Table 1) highlight the superiority of this method. Additionally, analyses of selectivity, repeatability, and resistance to interference (Fig. 3C and Fig. S5A and B) further affirm the robustness and reliability of this detection platform.

**Table 1.** Comparison of this proposed method with reported immunoassays for the detection of HIV-1 DNA

Techniques	Limit of detection	Detection range	Reference
Electrochemiluminescence	30 fM	100 fM–1,000 nM	[42]
Colorimetric assay	1.5 pM	10 pM–6.5 nM	[25]
Electrochemical sensor	3.8 fM	10 fM–10 pM	[43]
Fluorescence resonance energy transfer	15 fM	50 fM–1 nM	[44]
Fluorometric assay	5.3 pM	1–25 nM	[45]
Fluorometric assay	62 aM	100 aM–10 nM	This work

The effectiveness of our method was validated using HIV-1–positive clinical samples, with results demonstrating high consistency with those obtained via reverse transcription PCR (RT-PCR) (Fig. 3D). This alignment underscores the method’s reliability and potential for clinical application, highlighting its ability to accurately detect HIV biomarkers in real-world settings.

### Design principles of HIV-1 p24 detection

The detection principle for HIV-1 p24, as depicted in Fig. 1, builds on a synergistic workflow involving antigen–antibody binding, RCA, and CRISPR/Cas12a-mediated fluorescence signal generation. In this system, DNA-conjugated antibodies specifically bind to HIV-1 antigens, bringing probes P1′ and P2′ into close proximity on the antigen surface to facilitate proximity hybridization. This proximity triggers a series of reactions, starting with primer elongation, nicking, and dissociation, which generate primers that bind to padlock probes. These padlocks initiate RCA, producing long DNA products with repeating sequences that hybridize with crRNA. This hybridization activates the trans-cleavage activity of the Cas12a enzyme, leading to the cleavage of ssDNA reporters and the generation of a detectable fluorescent signal.

### Characterization and optimization of HIV-1 p24 detection

**Fig. 4.**
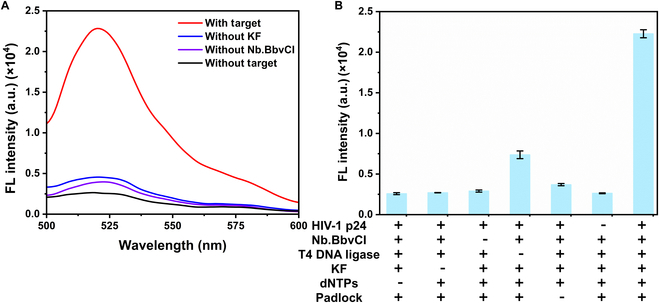
The feasibility was characterized by fluorescence signals. (A) From top to bottom: sample with target, sample with target and Nb.BbvCI but without KF, sample with target and KF but without Nb.BbvCI, and sample without target. (B) From left to right: sample without dNTPs, sample without KF, sample without Nb.BbvCI, sample without T4 DNA ligase, sample without padlock, sample without target, complete sample. Error bars mean SD, and every experiment was adopted 3 times.

The feasibility of this system was confirmed through fluorescence spectroscopy. High fluorescence signals were observed when the target, T4 DNA ligase, and Nb.BbvCI were present, while weak signals were detected in their absence (Fig. 4A). Multi-control experiments further validated the system’s reliability, demonstrating that insufficient levels of critical reagents, such as dNTPs, KF polymerase, Nb.BbvCI, padlocks, or targets, resulted in reduced fluorescence signals. Notably, the absence of T4 DNA ligase caused a marked reduction in fluorescence, likely due to incomplete cyclization of adjacent bases, although partial extension by KF polymerase still occurred (Fig. 4B). These results collectively confirm the robustness and specificity of the HIV-1 p24 detection system, highlighting its potential for accurate and sensitive detection of antigens in clinical and research applications.

### Analytical performance of HIV-1 p24 detection

**Fig. 5.**
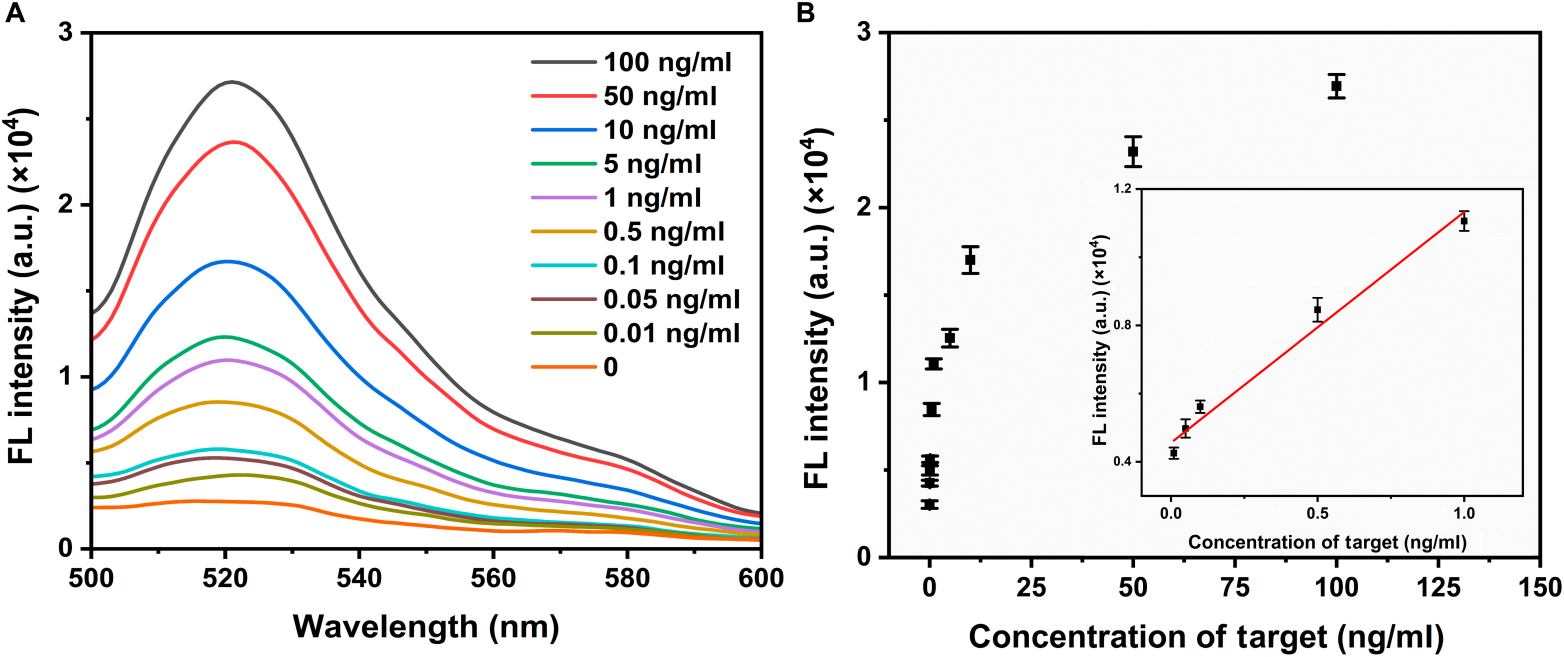
(A) Spectra of fluorescence at various concentrations of the target. (B) The correlation between efficiency of energy transfer through fluorescence and varying concentrations of HIV-1 p24. The inner illustration shows linear relationship between the fluorescence signal and the HIV-1 p24 concentration from 0.01 to 1 ng/ml. Error bars mean SD, and every experiment was adopted 3 times.

The detection system was optimized by modifying the buffer composition to enhance antibody and enzyme activity, ensuring reliable and robust performance. Among the various buffers tested, rCutSmart Buffer was identified as the most suitable due to its ability to minimize fluorescence errors while providing excellent system stability, as demonstrated in Fig. S6. This buffer was therefore selected for all subsequent experiments to achieve optimal reaction conditions. Optimal reaction times were identified as 2.5 h for the RCA system (Fig. S7A). Optimal concentration of padlock was identified as 250 nM (Fig. S7B). Optimal concentrations were determined to be 100 U/μl for T4 DNA ligase and 4 U/μl for KF polymerase (Fig. S7C and D). Sensitivity testing involved measuring fluorescence intensity across a wide range of target concentrations to assess the system’s ability to detect low-abundance antigens. As shown in Fig. 5A, fluorescence signals displayed a clear and proportional increase with rising target concentrations, reflecting the precision and dynamic range of the assay. The fitted curve at the 520-nm fluorescence peak exhibited a strong linear relationship between 0.01 and 1 ng/ml, described by the equation *y* = 6,769.500*x* + 4,558.588 with a high correlation coefficient (*R*^2^ = 0.974), where *x* represents the target concentrations and *y* represents the energy transferring efficiency index, as shown in Fig. 5B. The limit of detection (LOD) was calculated to be 8.48 pg/ml using the 3σ rule, highlighting the assay’s remarkable sensitivity compared to other biosensors for HIV-1 p24, as outlined in Table 2.

**Table 2.** Comparison of this proposed method with reported immunoassays for the detection of HIV-1 p24

Techniques	Limit of detection	Detection range	Reference
ELISA	204 pg/ml	26 pg/ml–2,000 ng/ml	[46]
Centrifugal microchannel array	5 pg/ml	5–80 pg/ml	[47]
Plasmonic nanochains	5 ng/ml	5–10 ng/ml	[48]
Photoelectrochemical immunoassay	10 ng/ml	10 ng/ml–100 μg/ml	[49]
Fluorometric assay	8.48 pg/ml	10 pg/ml–100 ng/ml	This work

**Fig. 6.**
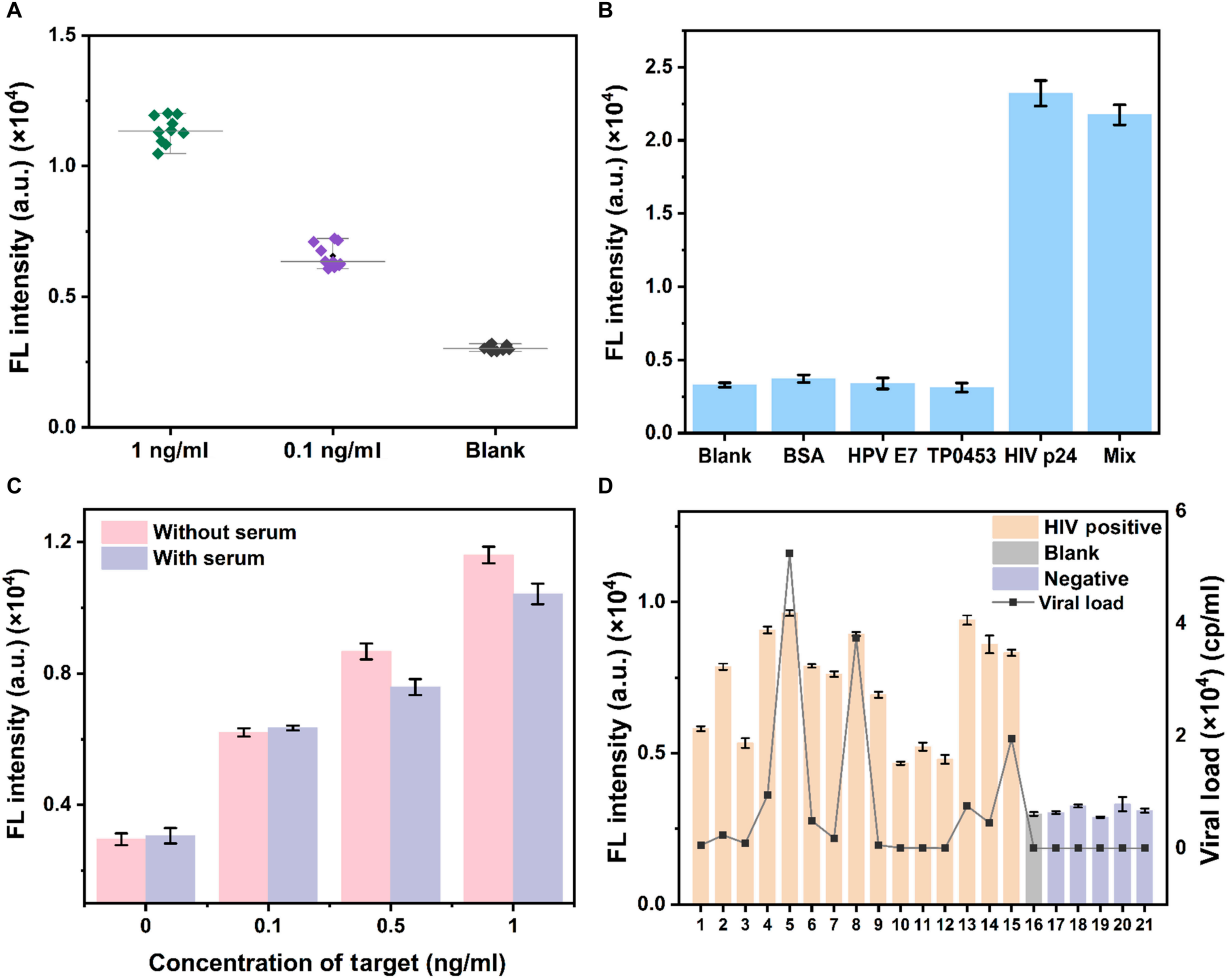
(A) Repeatability among 10 replicated experiments using samples with 1 ng/ml, 0.1 ng/ml, and blank of HIV-1 p24. (B) Specificities for different proteins [blank, bovine serum albumin (BSA), human papillomavirus protein E7 (HPV E7), *Treponema pallidum* outer membrane protein TP0453 (TP0453), HIV-1 p24, and a mixture (HIV-1 p24 + per interfering substance)]. (C) Comparison of different concentration of HIV-1 p24 (0, 20, 40, and 60 ng) prepared in water and fetal bovine serum (FBS). (D) Detection of clinical samples. *P* < 0.01. Error bars mean SD, and every experiment was adopted 3 times.

Repeatability testing was conducted using 10 independent replicates at both high and low target concentrations to verify the system’s reproducibility. The results, shown in Fig. 6A, demonstrated consistent performance across all replicates, with an inter-assay coefficient of variation below 7%, indicating the high reliability of the method. Selectivity was evaluated by introducing a variety of nontarget proteins at the same concentration to assess potential cross-reactivity. As illustrated in Fig. 6B, the system exhibited excellent specificity, with minimal signal interference from nontarget proteins, confirming its ability to accurately discriminate HIV-1 p24 antigens. Furthermore, the robustness of the assay was evaluated in the presence of complex biological matrices by introducing a control group containing 10% fetal bovine serum. As shown in Fig. 6C, the fluorescence signals across 4 target concentrations remained consistent regardless of the sample matrix, demonstrating the method’s strong resistance to interference and its suitability for use with real biological samples. To evaluate the accuracy of the HIV-1 p24 detection system, a double-blind parallel comparative study was conducted using ELISA as the reference method. The experimental design incorporated 7 randomly selected HIV-1 p24 antigen samples with varying concentrations and 3 target-negative control samples. As illustrated in Fig. S8, comparative analysis demonstrated that our method achieved accurate detection across all test samples, showing complete concordance with ELISA results.

The method’s clinical applicability was validated through the detection of HIV-1–positive clinical samples. Results obtained from the target-induced homogeneous detection closely aligned with those from RT-PCR, as shown in Fig. 6D, underscoring the method’s reliability and accuracy in real-world scenarios. This alignment highlights the potential of the proposed system as a powerful diagnostic tool for detecting HIV-1 p24 antigens, combining high sensitivity, specificity, and robustness in both controlled and complex sample environments. By addressing key challenges in antigen detection, such as cross-reactivity and matrix interference, this method provides a reliable and versatile platform for clinical and research applications, with the potential to significantly improve the diagnosis and management of HIV.

## Discussion

A versatile and innovative isothermal cascade amplification CRISPR/Cas12a assay has been developed, offering a universal platform for the detection of both genetic markers and proteins. This strategy is based on a generalizable approach that integrates 3 synergistic amplification stages: target-induced isothermal amplification, RCA, and reporter probe cleavage mediated by Cas12a. Extraordinary sensitivity has been achieved with this system, as demonstrated by its ability to detect HIV-1 DNA and HIV-1 p24 antigens, with detection limits of 62 aM and 8.48 pg/ml, respectively. Clinical samples were also taken, which meets the traditional clinical assays. These results highlight the assay’s capacity to detect extremely low-abundance targets, making it suitable for diverse applications in clinical diagnostics and molecular research.

The modular design of this assay allows for straightforward adaptation to various biomarkers by modifying the target-specific probes, thereby emphasizing its universal applicability. The need for separate detection platforms for different molecular targets is eliminated, and assay development for nucleic acids, proteins, and other biomolecules is streamlined. Robust signal amplification through RCA, combined with the programmable and sequence-specific cleavage activity of CRISPR/Cas12a, has enabled the creation of a highly sensitive and efficient detection system applicable to complex biological samples, including serum. Validation experiments have confirmed the system’s reliability in clinical sample analysis, with results shown to align closely with gold-standard RT-PCR methods, further demonstrating its practical utility.

Key limitations of existing diagnostic tools, such as limited sensitivity, complexity, and cost, are addressed by this approach. The elimination of thermal cycling requirements and the incorporation of isothermal amplification simplify the assay, making it suitable for deployment in resource-limited settings. The system’s flexibility and scalability position it as a valuable addition to the CRISPR diagnostics (CRISPR Dx) toolkit, enabling precise target identification and significant signal enhancement. Beyond HIV detection, this strategy is expected to provide a transformative impact in molecular diagnostics by serving as a universal framework for detecting a broad range of biomarkers, from infectious diseases to cancer markers.

The combination of high sensitivity, versatility, and simplicity positions this assay as a significant advancement in the field of molecular diagnostics. Its ability to bridge the gap between research and clinical applications ensures its adaptability to emerging diagnostic challenges. With its potential to expand the scope of CRISPR-based diagnostics, this system is poised to contribute significantly to personalized medicine, point-of-care testing, and global health initiatives.

The sensing platform developed in this study offers significant operational advantages, featuring a streamlined workflow that eliminates complex procedures such as washing steps while requiring no specialized technical expertise or bulky instrumentation. This system utilizes only essential reagents including buffer solutions and enzymes, substantially reducing material costs, with all necessary components being pre-fabricated for immediate use without preparatory procedures. The platform’s simplified design enables direct sample analysis while maintaining robust detection performance, demonstrating particular utility in resource-limited settings where conventional laboratory infrastructure may be unavailable.

## Materials and Methods

HIV-1 p24 antibodies (Ab1, Ab1) and HIV-1 p24 antigens were obtained from Xiamen Innobiomax Biotechnology Co. Ltd. The 1× EnGen Lba Cas12a Diluent, 10× NEBuffer 2.1 buffer, 10× NEBuffer r2.1, 10× rCutSmart Buffer, T4 DNA ligase, and nicking endonuclease (Nb.BbvCI) were obtained from New England Biolabs Inc. (USA). Deoxynucleotide solution mix (dNTPs) and Klenow fragment (3′- 5′exo-) were obtained from Beyotime Biotechnology (Shanghai, China). TCEP [tris(2-carboxyethyl)phosphine], DMF (*N*,*N*-dimethylformamide), and sulfo-SMCC [sulfosuccinimidyl 4-(*N*-maleimidomethyl)cyclohexane-1-carboxylate] were obtained from Shanghai Aladdin Biochemical Technology Co. Ltd. (Shanghai, China). All nucleic acid sequence synthesis in this study was provided by Sangon Biotech (Shanghai, China). All the nucleic acid sequences are listed in Table S1.

### HIV-1 DNA detection

To detect HIV-1 DNA, a reaction mixture was prepared by combining 1 μl of HIV-DNA, 1 μl of P1, 1 μl of P2, 1 μl of Klenow Fragment, Exo- Polymerase (2 U/μl), 1 μl of 10× reaction buffer, 1 μl of Nb.BbvCI enzymes (1 U/μl), 1 μl of 10× rCutSmart Buffer, 0.5 μl of dNTPs (2 mM), and 6.5 μl of 10× NEBuffer 2.1. The mixture was incubated for 45 min at 37 °C, followed by termination at 75 °C for 5 min. After this reaction, 1 μl of padlock, 1 μl of T4 DNA ligase (40 U/μl), and 1 μl of 10× T4 DNA ligase reaction buffers were added, and the mixture was incubated at 25 °C for 40 min. Then, 1 μl of Klenow Fragment, Exo- (2 U/μl), 1 μl of 10× reaction buffer, and 1 μl of dNTPs (2 mM) were added. The concentrations of P1, P2, and HIV-DNA were maintained at 25 nM, while the suspension probe was at 100 nM. This mixture was then incubated at 37 °C for 120 min and terminated at 75 °C for 5 min. Then, a pre-mixed CRISPR/Cas12a reaction system was added, consisting of 1 μl of crRNA (1 μM), 0.75 μl of Cas12a enzymes (1 μM), and 1.75 μl of 10× NEBuffer r2.1. This was followed by the addition of 1.5 μl of report probe (1 μM). The reaction was incubated for 40 min at 37 °C. Finally, 175 μl of ddH_2_O (double-distilled water) was added, resulting in a 200-μl product, which was then analyzed using fluorescent scanning. PAGE analysis was conducted to confirm the binding-induced isothermal amplification activity and RCA. A 15% polyacrylamide gel was prepared with 10× TBE buffer. The reaction products were diluted in a 6× loading buffer at a ratio of 5:1 for gel electrophoresis. Electrophoresis was carried out at 80 V for 100 min in a Tris–borate–EDTA (TBE) buffer solution. Gel staining was performed using Gel-Red Stain, and visualization was done using an ultraviolet (UV) transilluminator (Bio-Tanon, Shanghai, China).

### Preparation of DNA–antibody conjugates

DNA–antibody conjugates were prepared under dark conditions at 25 °C; 100 μl of Ab1 (1 mg/ml), 100 μl of Ab2 (1 mg/ml), and 40 μl of sulfo-SMCC (200 mM) in DMF were dissolved in phosphate-buffered saline (PBS) solution for 2 h. Simultaneously, 100 μl of P1′ (100 μM), 100 μl of P2′ (100 μM), and 100 μl of TCEP (40 mM) were then mixed and incubated for 1 h at 37 °C in ddH_2_O. Sulfo-SMCC-activated antibodies Ab1 and P1′, and Ab2 and P2′ were then mixed and incubated with magnetic stirring for 4 h at 25 °C. Unreacted DNA was removed by 72-h dialysis in PBS solution to obtain the DNA–antibody conjugates (Ab1–P1′, Ab2–P2′). Finally, the conjugations were purified by the 10K MWCO Zeba Spin Desalting Column and stored at 4 °C to avoid repeated freezing. The UV–vis absorption spectra of the antibody before and after conjugations were measured and are shown in Fig. S1.

### HIV-1 p24 detection

Firstly,1 μl of HIV-1 p24 antigen (1 ng/ml), 1 μl of Ab1–P1′, 1 μl of Ab2–P2′,1 μl of Klenow Fragment, Exo- (2 U/μl), 1 μl of 10× reaction buffer, 1 μl of Nb.BbvCI (1 U/μl), 7.5 μl of 10× rCutSmart Buffer, and 0.5 μl of dNTPs (2 mM) were mixed and reacted for 60 min at 37 °C. The reaction was ended at 75 °C for 5 min. After completion of the above reaction, the system was added with 1 μl of lock probes (250 nM), 1 μl of T4 DNA ligase (100 U/μl), and 1 μl of 10× T4 DNA ligase buffer (reaction at room temperature for 60 min). Then, 1 μl of Klenow Fragment, Exo- (2 U/μl), 1 μl of 10× reaction buffer, and1 μl of dNTPs (2 mM) were added to the abovementioned system after the connection reaction is completed. The system (20 μl) was incubated at 37 °C for 150 min. The reaction was ended at 75 °C for 5 min. Then, the pre-mixed CRISPR/Cas12a reaction system was added: 1 μl of crRNA (1 μM), 0.75 μl of Cas12a enzymes (1 μM), and 1.75 μl of 10× NEBuffer r 2.1, followed by 1.5 μl of report probe (1 μM), 40 min at 37 °C, and 175 μl of ddH_2_O after the reaction; the system generates 200 μl of product for fluorescent scanning and recording of data.

### Fluorescence measurement

The system’s performance was investigated using fluorescence spectrum analysis. The reaction products were stimulated with a 480-nm laser, and the resulting emission spectra were measured in the range of 500 to 600 nm.

### HRP-conjugated antibody preparation for ELISA

The conjugation of horseradish peroxidase (HRP) to antibodies was performed by adding 0.2 ml of HRP (10 mg/ml) to 0.2 ml of 0.06 M NaIO₄, followed by stirring at 4 °C in the dark for 30 min. Subsequently, 0.2 ml of ethylene glycol was added, and the mixture was stirred at room temperature in the dark for an additional 30 min. After the reaction, 2 mg of Ab2 antibody was introduced, and the pH was adjusted to 9.0, followed by continuous stirring at 4 °C in the dark for 24 h. NaBH₄ (0.08 ml, 5 mg/ml) was then rapidly added, and the solution was kept at 4 °C for 2 h. An equal volume of saturated (NH₄)₂SO₄ was slowly added, and the mixture was incubated at 4 °C for another 2 h before centrifugation to remove the supernatant. The resulting conjugate was dialyzed in the dark for 24 h and stored at −20 °C after mixing with an equal volume of glycerol.

### ELISA procedure

The coating antibody (Ab1) was diluted to 4 concentration gradients (0.3, 0.1, 0.03, and 0.01 μg/ml) and added to the microplate at 100 μl/well, followed by incubation at 37 °C for 2 h. After removing the coating solution, the wells were washed 3 times with 200 μl/well of washing buffer and dried by blotting on absorbent paper. Blocking was performed by adding 200 μl/well of blocking buffer and incubating at 37 °C for 2 h, followed by another washing step. Immune serum was diluted in antibody dilution buffer at ratios of 1:1,000, 1:3,000, 1:9,000, and 1:27,000, while 10 HIV p24 standard samples were diluted to appropriate concentrations using standard dilution buffer. A control group received 50 μl of 0.01 M PBS, whereas the experimental group was treated with an equal volume of standard dilution buffer, followed by the addition of diluted immune serum and incubation at 37 °C for 30 min before washing. HRP-Ab2 was diluted 3,000-fold in antibody dilution buffer and added to the 96-well plate at 100 μl/well, followed by incubation at 37 °C for 30 min and subsequent washing. Substrate solution (100 μl/well) was then added, and the plate was incubated at 37 °C for 20 min. The reaction was terminated by adding 50 μl/well of stop solution, and the optical density (OD) was measured at 450 nm using a microplate reader to complete the assay.

### HIV-1 DNA detection in clinical samples

Clinical samples used in this study were whole nucleic acid extraction solution derived from the blood of HIV-infected and healthy individuals, provided by Suzhou Center for Disease Control and Prevention.

### Proviral DNA extraction and detection

For HIV-1 DNA analysis, whole blood samples were processed using the QIAGEN EZ2 Connect automated nucleic acid extraction instrument coupled with the Virus Mini Kit v2.0. One microliter of the 10-fold diluted blood sample was added to the corresponding reaction solutions, following the same procedures as those used for buffer solutions. All experimental waste was properly inactivated after detections.

### HIV-1 p24 detection in clinical samples

Clinical samples used in this study were blood serum of HIV-infected and healthy individuals, provided by Suzhou Center for Disease Control and Prevention. One microliter of the 10-fold diluted blood sample was added to the corresponding reaction solutions, following the same procedures as those used for buffer solutions. All experimental waste was properly inactivated after detections.

### HIV-1 viral load quantification by RT-PCR

Viral RNA extraction was performed using the Roche COBAS AmpliPrep system. Subsequent quantification of HIV-1 was conducted on the Roche COBAS TaqMan analyzer with the Human Immunodeficiency Virus Type 1 Nucleic Acid Detection Kit (PCR fluorescence method), which utilizes TaqMan probe chemistry for real-time PCR amplification.

## Data Availability

Data will be made available on request.
