# Supplementary material for: A General Biosensing Strategy Based on Cascade Amplification for Enhanced HIV Detection Sensitivity
Source: BME Front. 2025 Jul 2;6:0139. doi: 10.34133/bmef.0139 (PMC12214299; doi:10.34133/bmef.0139)
Supplement: Supplementary 1 — Figs. S1 to S8 Table S1 [file bmef.0139.f1.docx]

Supplementary Material for A General Biosensing Strategy Based on Cascade Amplification for Enhanced HIV Detection Sensitivity

Yirui Zhang^1^^,2^, Jieyu Yan^2^, Wangheng Hou^3^, Qian Gao^4^, Tao Zhang^1,2^, Yan Gao^1,2^, Jingwen Li^1,2^, Kun Han^1,2^*

**Affiliations**

^1^School of Biomedical Engineering (Suzhou), Division of Life Sciences and Medicine, University of Science and Technology of China, Hefei, 230026, China

^2^CAS Key Lab of Bio-Medical Diagnostics, Suzhou Institute of Biomedical Engineering and Technology, Chinese Academy of Science, Suzhou, 215163, China

^3^NMPA Key Laboratory for Research and Evaluation of Infectious Disease Diagnostic Technology, School of Public Health, Xiamen University, Xiamen, 361102, China

^4^Suzhou Center for Disease Control and Prevention, Suzhou, 215004, China

*Address correspondence to: hank@sibet.ac.cn

**Table of Content:**

1. UV-vis absorption spectra the conjugations of the DNA and the antibody. (Figure S1)

2. 15% PAGE analysis of isothermal amplification and primer generation products and 15% PAGE analysis of RCA. (Figure S2)

3. Optimization of the number of complementary bases of P1 and P2. (Figure S3)

4. Optimization of HIV-1 DNA experimental conditions. (Figure S4)

5. Repeatability of HIV-1 DNA and comparison of different concentration of target DNA (Figure S5)

6. Optimization of the buffer system of experimental condition. (Figure S6)

7. Optimization of HIV-1 p24 experimental conditions. (Figure S7)

8. Results of HIV-1 p24 detection method compared with ELISA. (Figure S8)

9. DNA sequences used in this work. (Table S1)


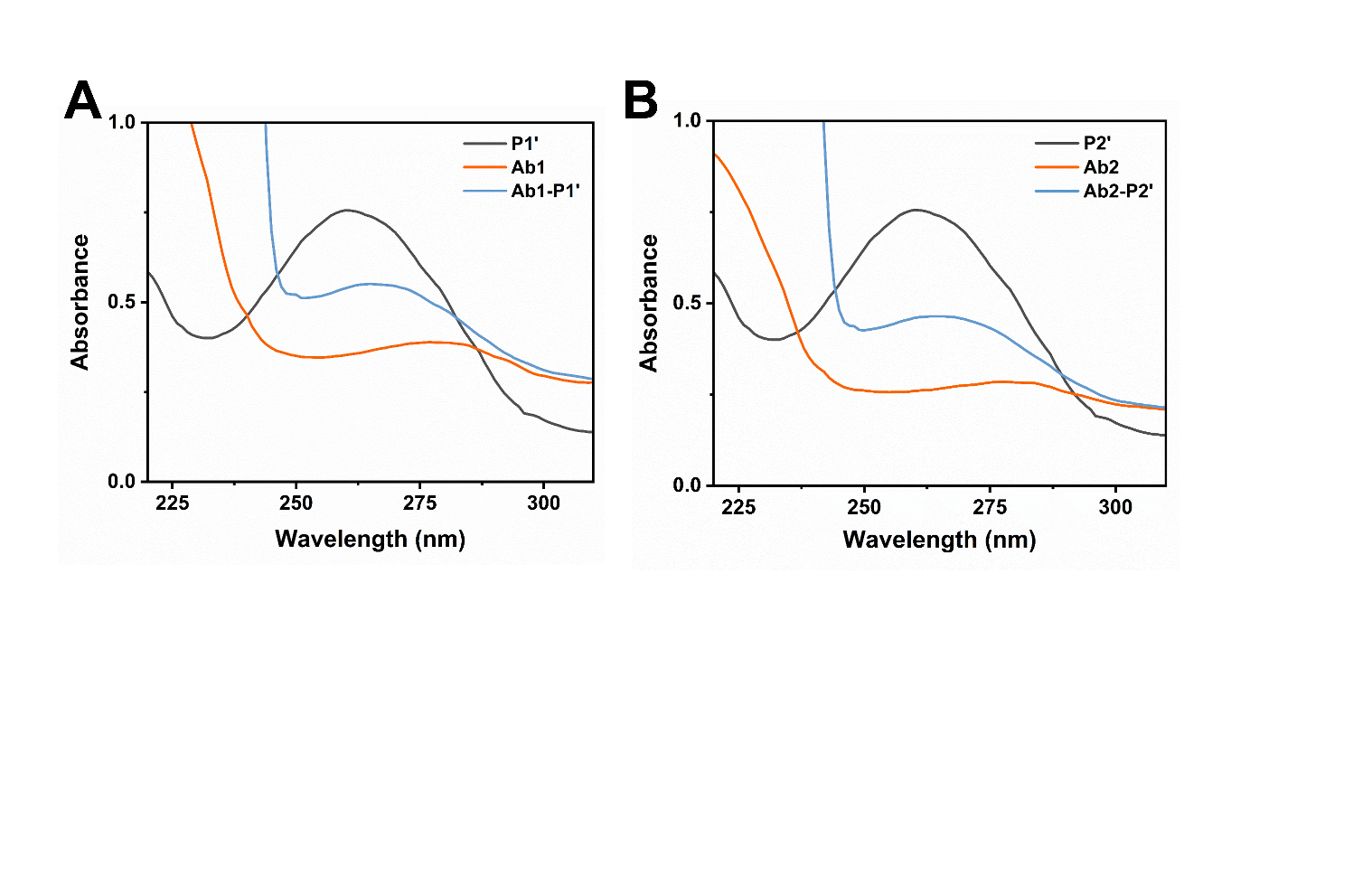


Fig S1. (A) UV-vis absorption spectra of Ab1-P1', Ab1 (1 mg/mL), P1' (100 μM). (B) UV-vis absorption spectra of Ab2-P2', Ab2 (1 mg/mL), P2' (100 μM). The spectrum of Ab-P1' shows a merged peak of the typical peak of DNA (260 nm) and the typical peak of antibody (280 nm), indicating the conjugations of the DNA and the antibody.


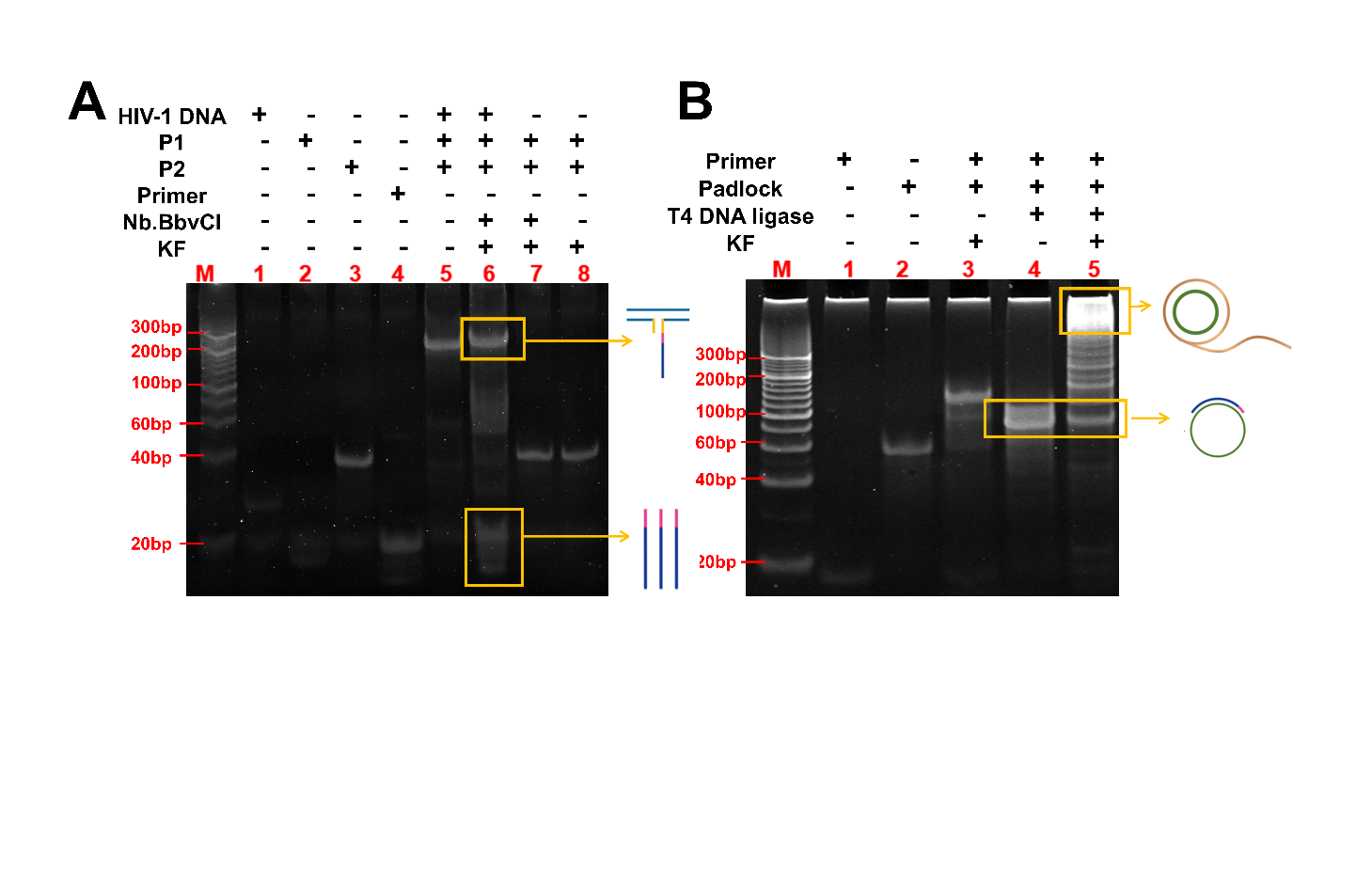


Fig S2. (A) 15% PAGE analysis of isothermal amplification and primer generation products. Lane 1: HIV-1 DNA, Lane 2: P1, Lane 3: P2, Lane 4: Primer, Lane 5: HIV-1 DNA+P1+P2, Lane 6: HIV-1 DNA+P1+P2+ Nb. BbvCI + KF, Lane 7: P1+P2+ Nb. BbvCI + KF, Lane 8: P1+P2+ KF. (B) 15% PAGE analysis of RCA. Lane 1: Primer, Lane 2: Panlock, Lane 3: Primer+Panlock+KF, Lane 4: Primer+Panlock+ T4 DNA ligase, Lane 5: Primer+Panlock+ T4 DNA ligase+KF.


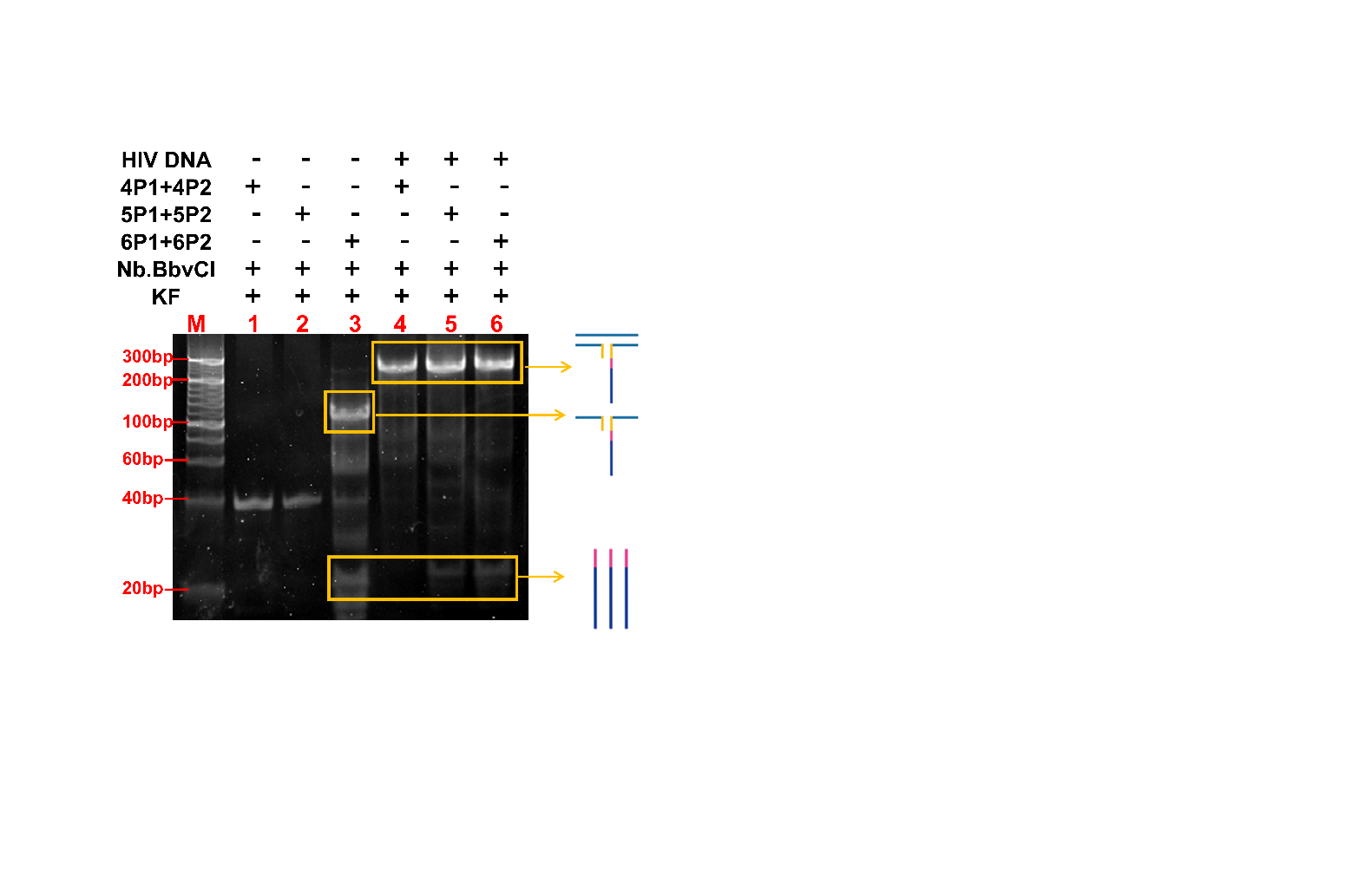


Fig S3. Optimization of the number of complementary bases of P1 and P2. Lane 1: 4P1+4P2+ Nb. BbvCI + KF, Lane 2: 5P1+5P2+ Nb. BbvCI + KF, Lane 3: 6P1+6P2+ Nb. BbvCI + KF, Lane 4: HIV-1 DNA+4P1+4P2+ Nb. BbvCI + KF, Lane 5: HIV-1 DNA+5P1+5P2+Nb. BbvCI +KF, Lane 5: HIV-1 DNA+6P1+6P2+ Nb. BbvCI + KF.


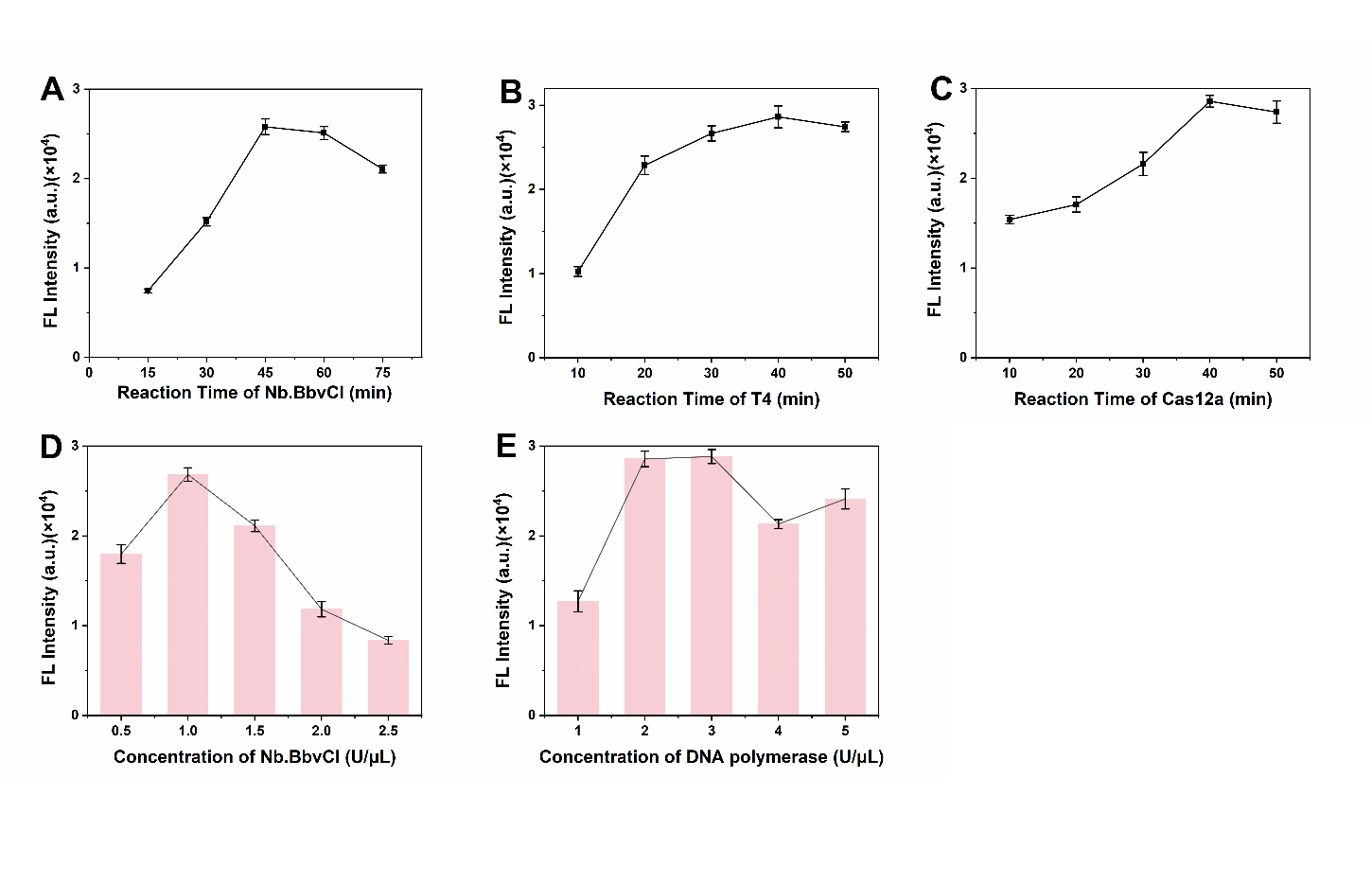


Fig S4. Optimization of HIV-1 DNA experimental conditions. (A)Effects of reaction time of Nb. BbvCI. (B) Effects of reaction time of T4 DNA ligase. (C) Effects of reaction time of the Cas12a system. (D) Effects of the concentration of Nb. BbvCI. (E) Effects of the concentration of KF in RCA. Error bars mean SD and every experiment was adopted three times.


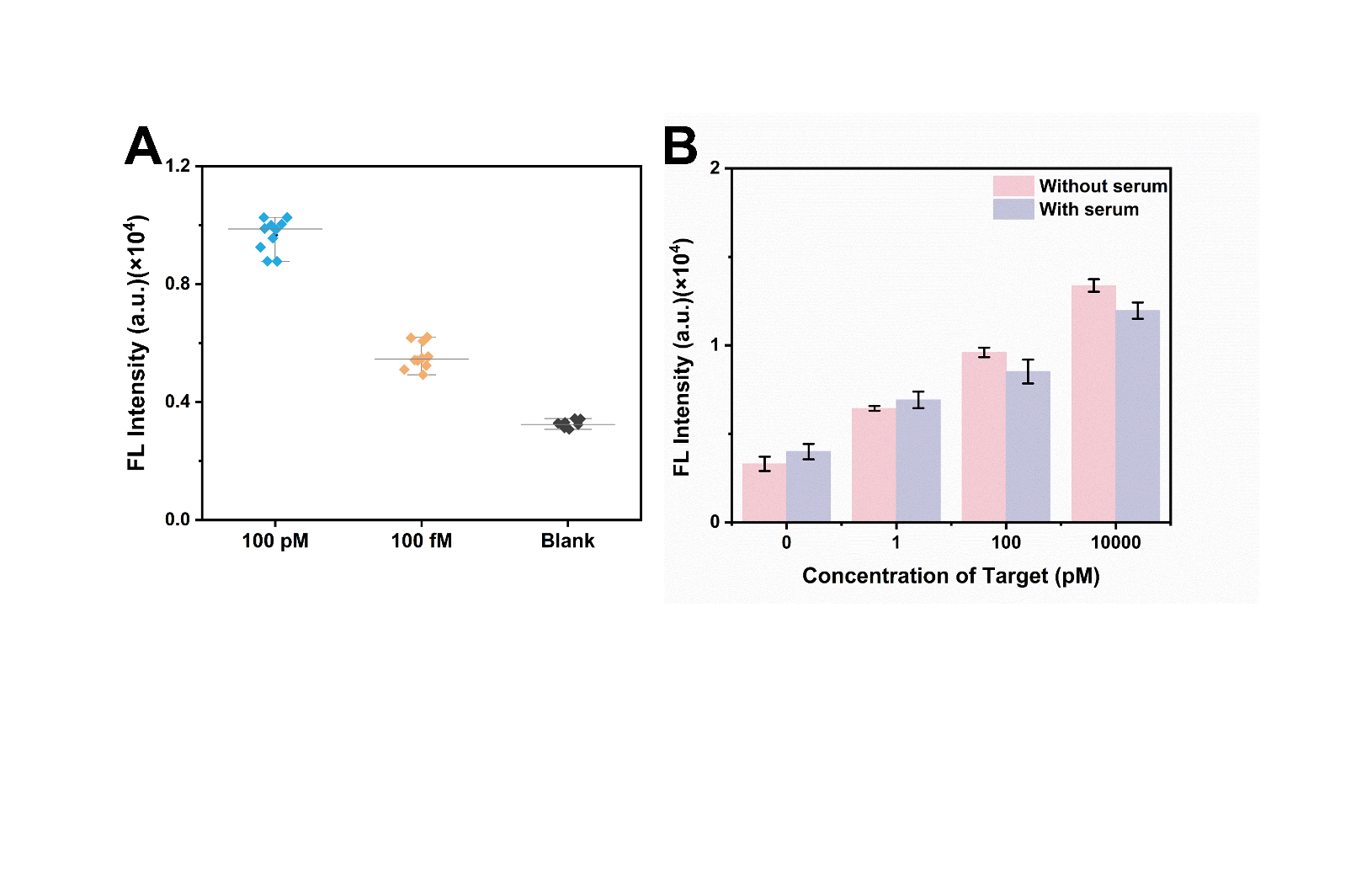


Fig S5. (A)Repeatability among 10 replicated experiments using samples with 100 pM, 100 fM and blank of HIV-1 DNA. (B) Comparison of different concentration of target DNA (0, 1,100 and 1000 pM) prepared in water and Fetal Bovine Serum (FBS). Error bars mean SD and every experiment was adopted three times.





Fig S6. Optimization of the buffer system of experimental condition. Error bars mean SD and every experiment was adopted three times.


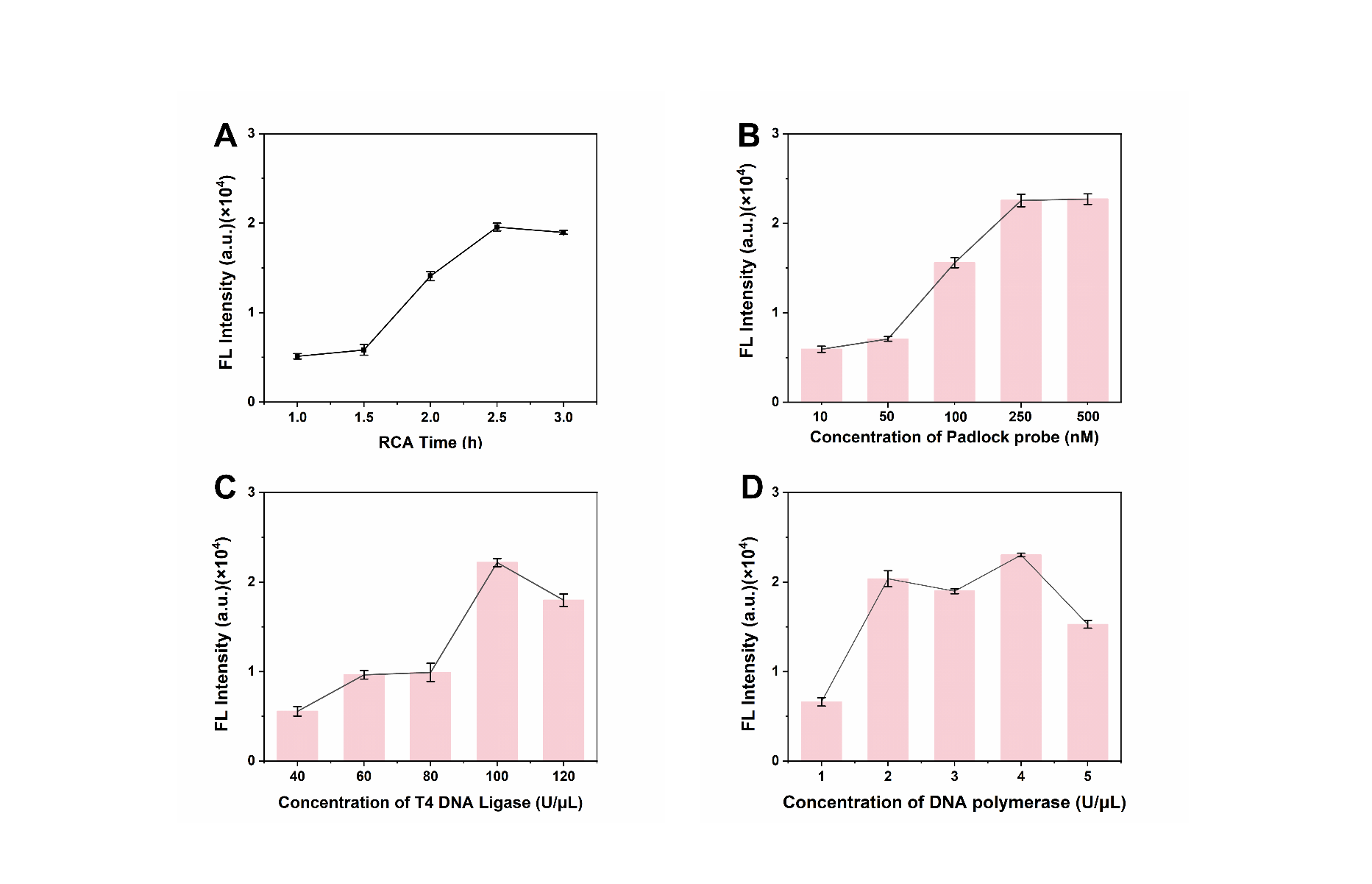


Fig S7. Optimization of HIV-1 p24 experimental conditions. (A) Effects of reaction time of RCA. (B) Effects of the concentration of padlock. (C)Effects of the concentration of T4 DNA ligase. (D) Effects of the concentration of KF in RCA. Error bars mean SD and every experiment was adopted three times.


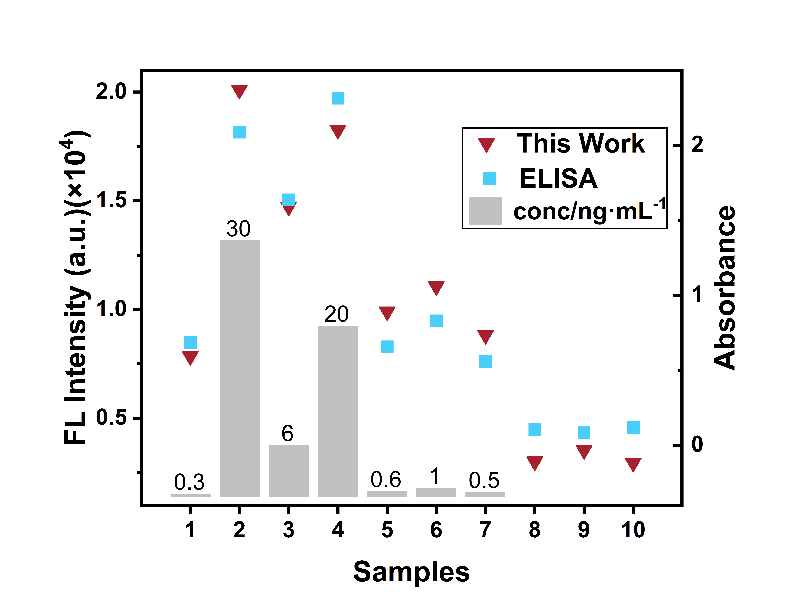


Fig S8. Results of HIV-1 p24 detection method compared with ELISA.

| HIV-1 DNA | GCTATACATTCTTACTATTTTATTTAATCCCAG |
| --- | --- |
| P1/5P1 | CTGGGATTAAATAAAATGCAACAC |
| P2/5P2 | CTCTAGTCTGACGTAATAGATCCTCAGCGTGTTAGTAAGAATGTATAGC |
| P1＇ | 5’HS-SH C6-TTTTTTTTTTTTTTTGAACAC |
| P2＇ | CTCTAGTCTGACGTAATAGATCCTCAGCGTGTTCTTTTTTTTTTTTTTT  -C6 HS-SH-3’ |
| Primer | TGAGGATCTATTACGTCAGACTAGAG |
| Padlock | 5’P-TAATAGATCCTCATAAAAAACAGCCACCAAGATCGCAAGTCTCATTTGT  GTACATCTCTAGTCTGACG |
| 6P1 | CTGGGATTAAATAAAATGCGAACAC |
| 6P2 | CTCTAGTCTGACGTAATAGATCCTCAGCGTGTTCAGTAAGAATGTATAGC |
| 4P1 | CTGGGATTAAATAAAATGCACAC |
| 4P2 | CTCTAGTCTGACGTAATAGATCCTCAGCGTGTAGTAAGAATGTATAGC |
| HCV DNA | GGCGACGCGGGATCCGACGTT |
| HPV DNA | AGTATTTTTATATGTAGTTTCTGAAGTAGATATG |
| PIK3CA E542K | CTCAGTGATTTTAGAGAGAGGAT |
| crRNA | UAAUUUCUACUAAGUGUAGAUCGCAAGUCUCAUUUGUGUACAU |
| Reporter probe | 5’6-FAM-TTATT-3’BHQ1 |

**Table S1.** DNA sequences used in this work.
